# Using average transcription level to understand the regulation of stochastic gene activation

**DOI:** 10.1098/rsos.211757

**Published:** 2022-02-16

**Authors:** Liang Chen, Genghong Lin, Feng Jiao

**Affiliations:** ^1^ Guangzhou Center for Applied Mathematics, Guangzhou University, Guangzhou, People’s Republic of China; ^2^ School of Mathematics and Information Sciences, Guangzhou University, Guangzhou, People’s Republic of China

**Keywords:** stochastic gene transcription, signalling transduction networks, mean transcription level, complex dynamics

## Abstract

Gene activation is a random process, modelled as a framework of multiple rate-limiting steps listed sequentially, in parallel or in combination. Together with suitably assumed processes of gene inactivation, transcript birth and death, the step numbers and parameters in activation frameworks can be estimated by fitting single-cell transcription data. However, current algorithms require computing master equations that are tightly correlated with prior hypothetical frameworks of gene activation. We found that prior estimation of the framework can be facilitated by the traditional dynamical data of mRNA average level *M*(*t*), presenting discriminated dynamical features. Rigorous theory regarding *M*(*t*) profiles allows to confidently rule out the frameworks that fail to capture *M*(*t*) features and to test potential competent frameworks by fitting *M*(*t*) data. We implemented this procedure for a large number of mouse fibroblast genes under tumour necrosis factor induction and determined exactly the ‘cross-talking *n*-state’ framework; the cross-talk between the signalling and basal pathways is crucial to trigger the first peak of *M*(*t*), while the following damped gentle *M*(*t*) oscillation is regulated by the multi-step basal pathway. This framework can be used to fit sophisticated single-cell data and may facilitate a more accurate understanding of stochastic activation of mouse fibroblast genes.

## 1. Introduction

**Figure 1.**
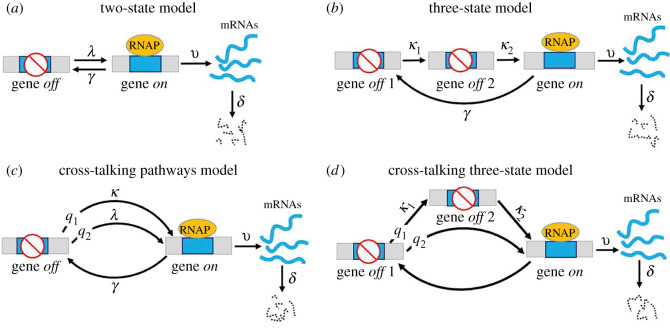
Different frameworks that direct stochastic gene activation (*on*). Other processes of gene inactivation (*off*), mRNA synthesis when the gene is *on*, and mRNA degradation are all determined by single rate-limiting steps at constant rates. (*a*) Two-state model. The gene is activated through a single rate-limiting step at a constant rate. (*b*) Three-state model. Two sequential rate-limiting steps regulate gene activation at a constant rate. (*c*) Cross-talking pathway model. The gene is activated by two different competitive rate-limiting pathways with selection probabilities *q*_1_ and *q*_2_ of the two pathways satisfying *q*_1_ + *q*_2_ = 1. (*d*) Cross-talking three-state model. The gene is activated either by a pathway with two sequential rate-limiting steps or a single rate-limiting pathway with constant rates.

Gene transcription is a random process in all genomic loci, wherein messenger RNA (mRNA) molecules for active genes are produced in a bursting fashion in which an episode of transcriptional activity is interrupted by irregular gene inactivation periods [1–3]. A central problem in studying stochastic gene transcription has been understanding the regulation scenarios that control random gene activation (*on*) and inactivation (*off*) in response to environmental changes [4–7]. The exponentially distributed *on* period is one of the few universal features of transcription present in both prokaryotic and eukaryotic genes [6,8,9]. However, the duration of *off* state is highly gene-specific, with its transition to *on* state being modelled as a single rate-limiting step (e.g. figure 1*a*) [1,3], a single pathway consisting of multiple sequential rate-limiting steps (e.g. figure 1*b*) [10], the parallel competitive rate-limiting pathways (e.g. figure 1*c*) [11] or a combination of both (e.g. figure 1*d*) [12]. These different gene activation frameworks, together with mRNA birth and death processes controlled by single rate-limiting steps, lead to different mathematical models (figure 1). These models have produced many important observations by fitting massive amounts of transcription data at the single-cell level [8,13,14].

Real-time imaging of transcriptional bursting makes it possible to count the durations of each gene *on* and *off* period along the entire timeline, which generates duration distributions for both gene *on* and *off* periods, respectively. For instances of the *Escherichia coli* P_lac/ara_ promoter [15] and yeast *FLO11* genes [16], their *on* and *off* periods are all well fitted by single exponential distributions. These observations support the classical two-state model shown in figure 1*a*, that the gene for turning genes *on* and *off* are all controlled by single rate-limiting biochemical steps [1,3]. However, the duration of the *off* state may vary with different genes, manifested by the observed distribution with a unique peak for mouse fibroblast genes [8] and *E. coli tetA* promoters [17]. The unimodal distribution of gene *off* duration can be mathematically explained by assuming gene activation frameworks in the three-state model (figure 1*b*), cross-talking pathways model (figure 1*c*) and cross-talking three-state model (figure 1*d*). However, these theoretical approaches cannot determine which framework is the best to describe the observed gene *off* distribution data, although they have provided good approximations to the downstream distribution of gene transcription [18,19].

The snapshot data for the distribution histogram of mRNA copy numbers in an isogenic cell population at different time points carry rich, dynamic information on the fluctuations in transcription [20]. When combined with mathematical models, the fit of mRNA (or other RNA types) distribution data has served as a powerful tool for revealing the multi-step regulation of the activation of different genes in bacteria, yeast and human cells [13,20,21]. However, the calculation of exact forms of dynamical mRNA distribution requires solving infinite arrays of chemical master equations under the whole parameter region of the models, which is beyond the scope of standard theoretical methods, even for the simplest two-state model (figure 1*a*) [22–24]. The fitting of mRNA distribution data must integrate several computational tools to determine the rate-limiting step numbers in the activation framework and search for suitable system parameters [13,20]. However, current computation algorithms focus only on a class of prior hypothetical multi-step gene activation [13,21,25]; therefore, additional competent frameworks may be ignored. Moreover, some typical dynamical transition patterns among mRNA distribution profiles can be well exhibited by different models [24,26–28], preventing a direct way to rule out models that do not panoramically match the transition patterns of dynamical mRNA distribution.

The steady-state measurement of gene transcription under different cellular conditions has generated a large dataset of mRNA distribution and its mean level *M*, the Fano factor *ϕ* (the variance over *M*), and noise *CV*^2^ (*ϕ* over *M*) [1,14]. Under mathematical models, fitting steady-state data has revealed a large spectrum of regulation scenarios that cells use in response to environmental changes [3,14,29]. The steady-state mRNA distributions observed so far can be typically classified into three modes [1]. However, the models in figure 1 can only generate the three distribution modes shown at steady state [10,23,30,31], suggesting that the limited mRNA distribution modalities are insufficient to map reversely onto the diversified frameworks of gene activation. The steady-state data of noise *CV*^2^, Fano factor *ϕ* and mean level *M*, when mapped as scattered points onto *M*-*CV*^2^ and *M*-*ϕ* planes, provide a diagram of trend lines of *CV*^2^ and *ϕ* against *M* under varying environments [2,14]. For a given gene of interest in *E. coli*, yeast or mammalian cells, the trend lines fitted by different models have revealed distinct regulation scenarios [32]. However, the scenario that plays a dominant role in gene regulation remains elusive.

In contrast to the time-consuming single-cell measurements that require RNA labelling and imaging with high sensitivity and resolution [15,21], the dynamical mRNA average level *M*(*t*) can be relatively easily captured by conventional methods at the cell population level [21,33]. Previous studies have revealed rich temporal profiles of *M*(*t*) for different genes and cellular conditions, such as monotonic increases in the *E. coli* promoter P_lac/ara_ [15], up-and-down behaviour in the *c-Fos* gene in human osteosarcoma [21], multiple peaks in mouse fibroblast genes [33,34], and even oscillations in yeast stress-induced genes [35]. These observations give rise to the problem of whether such rich dynamical behaviours of *M*(*t*) can be mapped back to the diversified frameworks of gene activation. The key objective is to establish bijections between the dynamical features of *M*(*t*) and the parameter regions for certain mathematical models. This allows us to rule out models that do not capture the exhibited dynamical features of *M*(*t*) and to test the simplest of the remaining models based on their fit to *M*(*t*) data. In this study, we assumed that gene activation is regulated by a combination of sequential and parallel pathways, as shown in figure 1, and we illustrated the way that dynamic mRNA average level data could be used to help estimate the gene activation frameworks.

## 2. Results

### 2.1. Cross-talking three-state model

To make the paper easier to follow, we focused on analysing the mRNA average level *M*(*t*) data from a large group of mouse fibroblast genes under cytokine tumour necrosis factor (TNF) stimulation conditions [33,34]. Except for the simple monotonic growth of *M*(*t*) generated by late response genes, the rich non-monotonic behaviours of *M*(*t*) have also been determined, such as up-and-down for the *Fos* gene, up-down-up for the *Cxcl1* gene, and damped oscillation with multiple peaks for the *Nfkbia* gene.

Theoretical bijections between the dynamical features of *M*(*t*) and three mathematical models were established (table 1) to determine the framework that can efficiently capture the rich transcription dynamics of mouse fibroblast genes. The two-state model shown in figure 1*a* can only generate monotonic increasing dynamics of *M*(*t*) [32] and thus is not suitable for the discussion of non-monotonic dynamical behaviours. The three-state model shown in figure 1*b* is proven to display damped oscillatory dynamics of *M*(*t*) under a certain parameter region [36,37]. However, such oscillation behaviour is almost invisible owing to its rapid exponential decay and only slightly decelerates the dynamic increase in *M*(*t*) [37]. The frameworks with two or more parallel pathways can capture the up-and-down dynamics of *M*(*t*) but fail to generate more complex transcription dynamics [32,38,39]. Moreover, the cross-talking pathway model (figure 1*c*) generates up-and-down *M*(*t*) only when the stronger pathway is frequently selected to activate the gene [32], which is incompatible with the robust up-and-down dynamics of *M*(*t*), even if the TNF induction level is extremely low [34]. Collectively, the activation frameworks of a single pathway or parallel pathways alone are insufficient to capture the rich dynamics of *M*(*t*) from mouse fibroblast genes (table 1).

**Table 1.** Bijection theory between *M*(*t*) dynamical features and system parameter regions for the (a) two-state [32], (b) three-state [36,37], (c) cross-talking pathways [32,38] and (d) multiple pathways models [39]. $\alpha ,\xi ,\Lambda ,\alpha_{1}$ and *x*_1_ are the auxiliary numbers associated with the system parameters, and *γ* and *δ* are the gene inactivation rate and mRNA degradation rate, respectively.

mathematical models	*M*(*t*) dynamical profiles	⟺	parameter regions
(a) two-state model	increase		all parameters
(b) three-state model	increase		*α*^2^ ≥ *ξ*
	almost increase^a^		*α*^2^ < *ξ*
(c) cross-talking pathways model	increase		Λ≥min{δ,γ}
	up-and-down		Λ<min{δ,γ}
(d) multiple pathways model	increase		*x*_1_ ≥ min{*δ*, *α*_1_}
	up-and-down		*x*_1_ < min{*δ*, *α*_1_}

^a^The three-state model generates a damped oscillatory *M*(*t*) when *α*^2^ < *ξ* [36,37]. However, such oscillation decays exponentially and displays visually increasing dynamics [37].

By combining the three-state model (figure 1*b*) and cross-talking pathways model (figure 1*c*), it is possible to generate new dynamic *M*(*t*) features. The simplest combination is shown in figure 1*d*, which we call the cross-talking three-state model. This model can be viewed as adding a parallel pathway in the three-state model or decomposing a pathway of the cross-talking pathways model into two sequential steps. We assumed that two competitive pathways activate the gene. These are the weak basal pathway, which has a selection probability *q*_1_ and consists of two sequential rate-limiting steps with strength rates *κ*_1_ and *κ*_2_, or the strong rate-limiting signalling pathway, which has a selection probability *q*_2_ and strength rate *λ*, satisfying

$$0<q_{1},q_{2}<1,\;q_{1}+q_{2}=1\;\text{and}\;0<\kappa_{1},\kappa_{2}<\lambda <\infty .$$

The basal pathway is regulated independently by a spontaneous mechanism to maintain basal transcription levels under normal cellular growth conditions [40,41]. The assumption of two sequential steps and low strength rates for the basal pathway is in close agreement with the real-time imaging data of the *off* period of 16 mouse fibroblast genes [8]. Moreover, suppose *κ*_1_ or *κ*_2_ is relatively large. In that case, the basal pathway can be mathematically approximated by a single rate-limiting step [36], and the framework in figure 1*d* reduces to the cross-talking pathways model (figure 1*c*). A stronger signalling pathway is triggered when cells receive external cues, and downstream transcription factors (TFs) are activated by special signal transduction pathways to upregulate gene transcription [34,42]. For each target gene, its activation is ultimately mediated through the binding of downstream TFs in the basal or signalling pathways at the cognate DNA sites in the gene promoter or enhancer domains [5,42]. The selection probabilities *q*_1_ and *q*_2_ may then quantify the concentration and availability of activated TFs in each pathway to competitively form TF/DNA binding configurations while the inducible activation rate *λ* of the signalling pathway quantifies the binding accessibility and strength between the corresponding TFs and DNA sites [5,12,39,42].

### 2.2. Dynamics of *M*(*t*) and the bijection with parameter regions

To establish the bijection between the *M*(*t*) profiles and the parameter regions of the model (figure 1*d*), we first need to calculate the exact forms of *M*(*t*) in terms of system parameters. At time *t* ≥ 0, let random variable *X*(*t*) = *X* = *o*_11_, *o*_12_, *o*_2_, *e*, specify the states of gene *off* 1 for basal pathway, gene *off* 1 for signalling pathway, gene *off* 2, and gene *on*, respectively. Then define

$$P_{m,X}(t)=\text{Prob}\{\text{the system is residing at state}\;X\;\text{with}\;m\;\text{mRNA molecules at time}\;t\},$$

and the mass function

$$P_{m}(t)=P_{m,o_{11}}(t)+P_{m,o_{12}}(t)+P_{m,o_{2}}(t)+P_{m,e}(t),\;m=0,1,\ldots$$

that quantifies the probability of *m* mRNA transcripts at time *t* in a single cell.

Following the standard procedure, we can obtain an infinite array of master equations with respect to *P*_*m*,*X*_(*t*) [10,36,43]

2.1 $$\;P_{m,o_{11}}^{\prime }(t)=-(\kappa_{1}+m\delta )P_{m,o_{11}}(t)+(m+1)\delta P_{m+1,o_{11}}(t)+q_{1}\gamma P_{m,e}(t),$$

2.2 $$\;P_{m,o_{12}}^{\prime }(t)=-(\lambda +m\delta )P_{m,o_{12}}(t)+(m+1)\delta P_{m+1,o_{12}}(t)+q_{2}\gamma P_{m,e}(t),$$

2.3 $$\;P_{m,o_{2}}^{\prime }(t)=-(\kappa_{2}+m\delta )P_{m,o_{2}}(t)+(m+1)\delta P_{m+1,o_{2}}(t)+\kappa_{1}P_{m,o_{11}}(t),$$

2.4 $$\begin{array}{rl} P_{m,e}^{\prime }(t) & =-(\gamma +v+m\delta )P_{m,e}(t)+(m+1)\delta P_{m+1,e}(t)+vP_{m-1,e}(t)+\kappa_{2}P_{m,o_{2}}(t)\\ & \;+\lambda P_{m,o_{12}}(t). \end{array}$$

Summing up (2.1)–(2.4) gives the master equation of *P*_*m*_(*t*)

2.5 $$P_{m}^{\prime }(t)=(m+1)\delta P_{m+1}(t)-vP_{m,e}(t)-m\delta P_{m}(t)+vP_{m-1,e}(t).$$

We did not focus on solving (2.1)–(2.5), which are beyond the scope of current mathematical methods within all parameter regions [22–24,26–28]. However, the master equations set a basis for calculating analytical forms for the gene state probabilities $P_{o_{11}},P_{o_{12}},P_{o_{2}},P_{e}$, and mean transcript level *M*(*t*), defined as

$$P_{X}(t)=\sum_{m=0}^{\infty }P_{m,X}(t),X=o_{11},o_{12},o_{2},o_{e}\;\text{and}\;M(t)=\sum_{m=0}^{\infty }mP_{m}(t).$$

Using these definitions and (2.1)–(2.5), we derived the equations for the four state probabilities and the mean transcription level

2.6 $$\begin{array}{rl} P_{o_{11}}^{\prime }(t) & =-\kappa_{1}P_{o_{11}}(t)+q_{1}\gamma P_{e}(t),\;P_{o_{12}}^{\prime }(t)=-\lambda P_{o_{12}}(t)+q_{2}\gamma P_{e}(t),\\ P_{o_{2}}^{\prime }(t) & =\kappa_{1}P_{o_{11}}(t)-\kappa_{2}P_{o_{2}}(t),\;P_{e}^{\prime }(t)=\lambda P_{o_{2}}(t)+\kappa_{2}P_{o_{2}}(t)-\gamma P_{e}(t)\\ \text{and}\;\;\;\;M^{{\;}^{\prime }}(t) & =vP_{e}(t)-\delta M(t). \end{array}\}$$

Because the system must reside on exactly one gene state at any time, we set an arbitrary initial condition for (2.6)

2.7 $$P_{o_{11}}(0)+P_{o_{12}}(0)+P_{o_{2}}(0)+P_{e}(0)=1\;\text{and}\;M(0)\geq 0.$$

We utilized the Laplace transform method to solve the first-order differential system (2.6) and (2.7) (electronic supplementary material). We defined a polynomial function as

$$\begin{array}{rl} h(x) & =[(\kappa_{1}\lambda +\kappa_{2}\lambda +\kappa_{1}\kappa_{2})P_{e}(0)+\kappa_{1}\kappa_{2}P_{o_{11}}(0)+\lambda (\kappa_{1}+\kappa_{2})P_{o_{12}}(0)+\kappa_{2}(\lambda +\kappa_{1})P_{o_{2}}(0)]x\\ & \;+P_{e}(0)x^{3}+[(\kappa_{1}+\lambda +\kappa_{2})P_{e}(0)+\lambda P_{o_{12}}(0)+\kappa_{2}P_{o_{2}}(0)]x^{2}+\kappa_{1}\kappa_{2}\lambda , \end{array}$$

and two auxiliary numbers *c*_1_ and *c*_2_ in terms of the system parameters (electronic supplementary material, (7) and (8)). It can be verified that zero is a simple eigenvalue of the coefficient matrix for the system of the first four equations in (2.6). The other non-zero eigenvalues *a*_1_, *a*_2_ and *a*_3_ are calculated in terms of *c*_1_, *c*_2_ and system parameters (electronic supplementary material, (4)–(6)). Under the arbitrary initial condition (2.7), the average mRNA level *M*(*t*) is found to be as follows:

(1) If $c_{1}^{2}<c_{2}$, *a*_1_, *a*_2_ and *a*_3_ are real numbers with 0 < *a*_1_ < *a*_2_ < *a*_3_ (electronic supplementary material, (11)), and *M*(*t*) takes the form of
  2.8 $$\begin{array}{rl} M(t) & =\frac{v}{\delta }\frac{\kappa_{1}\kappa_{2}\lambda }{a_{1}a_{2}a_{3}}-\frac{vh(-a_{1})e^{-a_{1}t}}{a_{1}(a_{2}-a_{1})(a_{3}-a_{1})(\delta -a_{1})}\\ & \;+\frac{vh(-a_{2})e^{-a_{2}t}}{a_{2}(a_{2}-a_{1})(a_{3}-a_{2})(\delta -a_{2})}-\frac{vh(-a_{3})e^{-a_{3}t}}{a_{3}(a_{3}-a_{1})(a_{3}-a_{2})(\delta -a_{3})}\\ & \;+\frac{vh(-\delta )e^{-\delta t}}{\delta (\delta -a_{1})(\delta -a_{2})(\delta -a_{3})}+M(0)e^{-\delta t}. \end{array}$$
(2) If $c_{1}^{2}=c_{2}$, *a*_1_, *a*_2_, *a*_3_ > 0 are real numbers with *a*_1_ ≠ *a*_2_ = *a*_3_ (electronic supplementary material, (13)), and *M*(*t*) takes the form of
  2.9 $$\begin{array}{rl} M(t) & =\frac{v}{\delta }\frac{\kappa_{1}\kappa_{2}\lambda }{a_{1}a_{2}^{2}}(1-e^{-a_{2}t})-\frac{vh(-a_{1})(e^{-a_{1}t}-e^{-a_{2}t})}{a_{1}(\delta -a_{1}){\left(a_{2}-a_{1}\right)}^{2}}+\frac{vh(-a_{2})te^{-a_{2}t}}{a_{2}(a_{2}-a_{1})(\delta -a_{2})}\\ & \;+\frac{vh(-\delta )(e^{-\delta t}-e^{-a_{2}t})}{\delta (\delta -a_{1}){\left(\delta -a_{2}\right)}^{2}}+M(0)e^{-\delta t}. \end{array}$$
(3) If $c_{1}^{2}>c_{2}$, *a*_1_ > 0 is a real number, whereas *a*_2_ and *a*_3_ are conjugate complexes, then let *a*_*r*_ = Re(*a*_2_) and *a*_*i*_ = Im(*a*_2_) (electronic supplementary material, (15)). Then, *M*(*t*) takes the form of
  2.10 $$\begin{array}{rl} M(t) & =\frac{v}{\delta }\frac{\kappa_{1}\kappa_{2}\lambda }{a_{1}(a_{r}^{2}+a_{i}^{2})}-\frac{vh(-a_{1})e^{-a_{1}t}}{a_{1}(\delta -a_{1})[{\left(a_{1}-a_{r}\right)}^{2}+a_{i}^{2}]}+\frac{vh(-\delta )e^{-\delta t}}{\delta (\delta -a_{1})[{\left(\delta -a_{r}\right)}^{2}+a_{i}^{2}]}\\ & \;+\bar{A}\sqrt{1+A^{2}}\cos(a_{i}t+\theta )e^{-a_{r}t}+M(0)e^{-\delta t}, \end{array}$$
  where the constants $\bar{A},A$ and *θ* correlate with the parameters and initial conditions (electronic supplementary material, (17)).

We characterized the dynamic profiles of *M*(*t*) with almost no expression products at the initial time *t* as in the experiments of [15,21,33]. We assumed that transcription starts from the gene *off* 1 state and counts only the newly produced mRNA molecules. This gives the following initial values:

2.11 $$(P_{o_{11}}(0),P_{o_{12}}(0),P_{o_{2}}(0),P_{e}(0),M(0))=(q_{1},q_{2},0,0,0).$$

We started with an interesting case, $c_{1}^{2}>c_{2}$. Then, *M*(*t*) was expressed in exact form (2.10), which contained a cosine function, and suggested possible oscillatory dynamics of *M*(*t*). However, the coefficient of the cosine function damped exponentially, which drastically weakened the oscillation visually, as manifested by our numerical examples and observations from the three-state model [37]. The exact form (2.10) can only be viewed as a steady-state value, adding a maximum of three exponential functions. If the exact form of *M*(*t*) with *M*(0) = 0 has such a structure, it can be proved that the most complex dynamics of *M*(*t*) will only take a unique peak such as up-and-down or up-down-up profiles (appendix theorem A.1).

For the other case of $c_{1}^{2}\leq c_{2}$, the eigenvalues *a*_1_, *a*_2_ and *a*_3_ are real, and the exact forms (2.8) and (2.9) of *M*(*t*) do not contain oscillatory functions but contain multiple exponential functions. The case $c_{1}^{2}=c_{2}$ rarely occurs in biology and can be viewed mathematically as a limiting case of $c_{1}^{2}<c_{2}$. For $c_{1}^{2}<c_{2}$, a rigorous statement of the *M*(*t*) profiles is inevitably technical. Let *x*_1_ and *x*_2_ denote the two roots of

$$H(x)=vq_{2}\lambda x^{2}+v(q_{1}\kappa_{1}\kappa_{2}+q_{2}\lambda \kappa_{1}+q_{2}\lambda \kappa_{2})x+v\kappa_{1}\kappa_{2}\lambda .$$

When both *x*_1_ and *x*_2_ are complex numbers, there are a total of four parameter correlations, and we showed that *M*(*t*), expressed by (2.8), either increases monotonically for all *t* > 0, or develops an up-down-up profile. If *x*_1_ and *x*_2_ are real values, we can classify all 60 correlations among *x*_1_, *x*_2_, *a*_1_, *a*_2_, *a*_3_ and *δ* into three categories that correspond to three distinct dynamical behaviours: the increasing, up-and-down and up-down-up profiles of *M*(*t*). We illustrated detailed mathematical results and their proof in appendix theorem A.2. In summary, for the cross-talking three-state model (figure 1*d*), even if *M*(*t*) is expressed in different exact forms (2.8)–(2.10) and influenced by various parameter correlations, *M*(*t*) can exhibit, but exhibits only three features: increasing, up-and-down and up-down-up dynamics.

### 2.3. Fitting dynamical transcription data of mouse fibroblast genes

**Figure 2.**
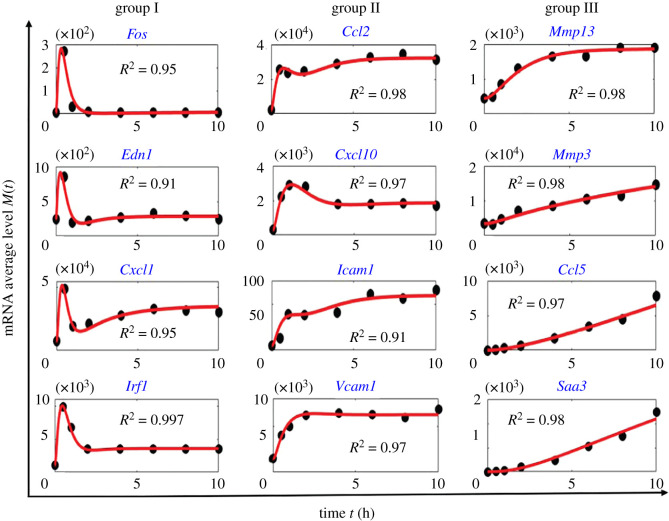
Fit of transcription data by the cross-talking three-state model (figure 1*d*). Black circles represent the dynamical data of mRNA average levels for 12 mouse fibroblast genes under TNF induction [33]. The genes are divided into groups I, II and III, which are separately characterized by the short, median and long half-lives of the transcripts [33]. The red lines are generated using exact forms (2.8)–(2.10) of the model (figure 1*d*), which provide a good fit to the data points (*R*^2^ > 0.9). The fitted system parameters are listed in electronic supplementary material, table S1.

We demonstrated three dynamical profiles of *M*(*t*) generated by the cross-talking three-state model (figure 1*d*). These distinct behaviours correspond with the observed dynamic trends of average mRNA levels in mouse fibroblast genes in response to TNF [33,34]. For instance, Hao & Baltimore [33] divided 180 activated mouse fibroblast genes under TNF induction into three groups, separately characterized by the short, median and long half-lives of the transcripts. As shown in figure 2, they found that the group I genes responded quickly by forming a sharp dynamical peak at average transcription levels; group II genes did not respond quickly. However, most still formed a gentle transcription peak along the timeline, and group III mRNAs accumulated rather slowly and gradually increased in abundance during the observation window. The transcription data from the 12 representative mouse fibroblast genes shown in figure 2 contains four genes displaying the up-down-up trend of transcription dynamics (*Edn1, Cxcl1, Ccl2, Icam1*), as well as others displaying either dynamical up-and-down or monotonic increasing transcription.

The fit of *M*(*t*) data in the case of measurement noise can be achieved by minimizing weighted least squares *J*(*θ*) with parameter values *θ*, an objective function indicating the difference between experiment and simulation [44]. For the sake of simplicity, we assumed a standard normally distributed measurement noise and then *J*(*θ*) is given by

$$J(\theta )=\sum_{\;j=1}^{N}{\left(M_{j}-M(t_{j},\theta )\right)}^{2}.$$

Here *N* is the number of measurement time points, *M*_*j*_ is the experimental data at time *t*_*j*_, and *M*(*t*_*j*_, *θ*) is the simulated *M*(*t*) at *t* = *t*_*j*_ obtained by using exact forms (2.8)–(2.10), or by the efficient computational methods on master equations (2.1)–(2.4) [25] or ordinary differential equations (2.6) and (2.7) [44]. To reduce the dimension of parameter space, we assigned extremely small values to the activation rates *κ*_1_ and *κ*_2_ of the weak basal pathway. The initial values of the other parameters that initialize the minimization runs of *J*(*θ*) are randomly sampled as in [44]. As shown in figure 2, the cross-talking three-state model provides good theoretical fits to all 12 datasets (coefficient of determination *R*^2^ > 0.9). We performed dynamical sensitivity analysis of *M*(*t*) with respect to the fitted parameters using forward finite differences [44] (electronic supplementary material, figure S1). It shows that the combination of *δ*, *q*_2_ and *γ* significantly influences increasing dynamics of *M*(*t*) for group III genes, while the variation of *q*_2_ plays a dominant role on influencing non-monotonic *M*(*t*) dynamics for groups I and II genes.

**Figure 3.**
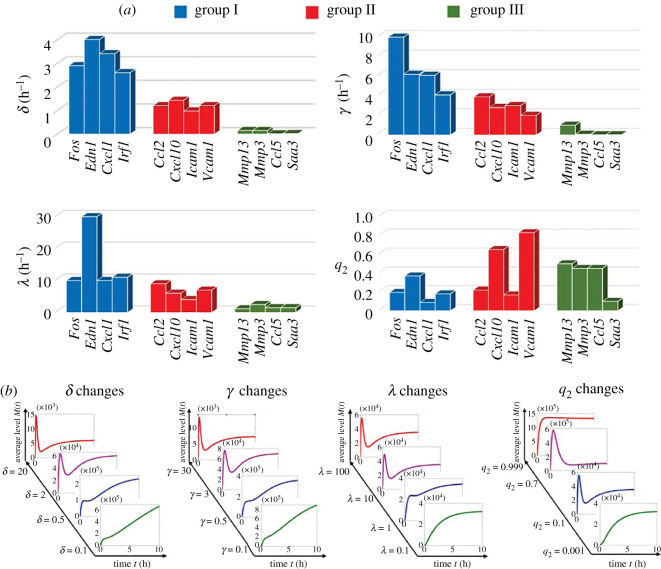
Regulation of transcription dynamics by system parameters. (*a*) Fitted parameters for 12 mouse fibroblast genes are listed in units of gene groups (electronic supplementary material, table S1). Different gene groups exhibit distinct temporal transcription modes [33]. The values of mRNA degradation rate *δ*, inactivation rate *γ*, and activation rate *λ* of the signalling pathway are all negatively correlated with gene groups I, II and III. The probability *q*_2_ of the signalling pathway does not follow a clear correlation with the gene groups. (*b*) Based on the fitted parameter set for the *Cxcl1* gene, increasing *δ*, *γ* and *λ* transit transcription dynamics from monotonic to up-down-up mode. An increase in *q*_2_ generates switches among multiple transcription dynamical modes, where the non-monotonic modes are displayed within most of the *q*_2_ variation region (0, 1).

While fitting the data shown in figure 2, we found that the estimated mRNA degradation rate *δ* of each gene falls within the *δ* region of the gene group to which they belong [33] (electronic supplementary material, table S1). Therefore, the fitted *δ* values exhibited a significant negative correlation with gene groups I, II and III because the gene groups themselves were classified by their transcript half-lives (figure 3*a*). Intriguingly, the freely fitted inactivation rate *γ* and activation rate *λ* of the signalling pathway also exhibited a negative correlation with gene groups (figure 3*a*). This observation suggests that the simultaneous large *λ* and *γ* may separately help in increasing the height of the peak and suppressing the stationary values of *M*(*t*) in group I. By contrast, the small *λ* and *γ* implemented contrary functions that destroy the dynamical peak of *M*(*t*) and lift the stationary mRNA numbers in group III. However, the probability *q*_2_ of the signalling pathway did not correlate with the gene groups but varied for different genes (figure 3*a*). Note that the dynamics of *M*(*t*) in the three gene groups are primarily discriminated by its first peak. Our observations suggest that, once cells receive external cues, the frequency of the signalling pathway directing gene activation may not play a crucial role in regulating the dynamical peak of transcription level.

To further understand the regulation of *M*(*t*) profiles, we varied the parameters *δ*, *γ*, *λ* and *q*_2_ under the fitted parameter sets of all 12 genes in figure 2. This procedure reveals a uniform regulation mode for each system parameter. As shown in figure 3*b* for *Cxcl1* gene, the variation in *δ* behaves as a bilateral switch to regulate *M*(*t*): there is a threshold value such that *M*(*t*) increases monotonically. By contrast, *δ* stays below the threshold but switches to a non-monotonic profile once *δ* exceeds the threshold. Such bilateral regulation of *δ* has been observed to play an important role in controlling the temporal transcription mode in mouse fibroblasts [33,34]. In addition, both *γ* and *λ* play the same bilateral roles in regulating *M*(*t*) dynamics (figure 3*b*), reinforcing previous observation of smaller *δ*, *γ*, *λ* for group III genes that generate increasing *M*(*t*) with larger *δ*, *γ*, *λ* for groups I and II genes that exhibit non-monotonic transcription dynamics (figure 3*a*). The regulation scenario of *q*_2_ is different because it generates multiple switches among distinct *M*(*t*) profiles, when *q*_2_ increases from 0 to 1 (figure 3*b*). Exceptions need to be made for cases where *q*_2_ approaches 0 or 1, which generates increasing transcription dynamics, because the dynamical peak of *M*(*t*) seems to be robust in almost all the various regions of *q*_2_ ∈ (0, 1). Note that *q*_2_ is closely related to signal strength. Our observations fit with the ubiquitous transcription dynamical peak of mouse fibroblast genes under TNF induction from the lowest to the highest levels [34].

### 2.4. Cross-talking *n*-state model for oscillatory transcription dynamics

**Figure 4.**
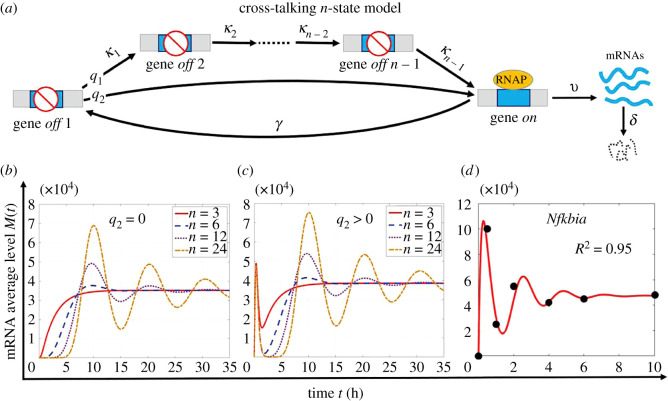
Framework for generating oscillatory transcription dynamics. (*a*) Cross-talking *n*-state model for which the gene is activated either by the strong signalling pathway with probability *q*_2_, or by the weak basal pathway consisting of *n* − 1 rate-limiting steps with probability *q*_1_ = 1 − *q*_2_. (*b*) When gene activation is directed by a single multi-step pathway (*q*_2_ = 0), the increase in the step number prolongs the initial response lag. In addition, it enhances the damped oscillation of the transcription level *M*(*t*). (*c*) When the gene is activated by the cross-talk between pathways (*q*_2_ > 0), the increase in the step number in the basal pathway has almost no impact on the initial quick up-and-down dynamics of *M*(*t*), but significantly enhances the following damped oscillatory behaviour. (*d*) Curve of *M*(*t*) (red lines) generated by the framework in (*a*) captures the oscillatory trend of transcription data (black circles) for the mouse fibroblast *Nfkbia* gene under TNF induction [33]. The parameters in (*b*) and (*c*) are the fitted rates of the *Cxcl1* gene in electronic supplementary material, table S1 with *κ*_1_ = · · · = *κ*_*n*−1_ to maintain a constant *T*_off_. Parameters in (*d*) are listed in electronic supplementary material, table S1.

Our bijection theory shows that the cross-talking three-state model (figure 1*d*) cannot generate multiple dynamical peaks of *M*(*t*). Therefore, the model (figure 1*d*) can be ruled out when *M*(*t*) exhibits oscillation [33,35]. We focused on transcription with constant kinetic rates under stable inductions to avoid complicated *M*(*t*) dynamics generated by time-dependent rates under time-varying signals [45] or feedback regulations [46]. The bijection theory (table 1) shows that multiple parallel pathways induce at most one dynamical peak, and therefore introducing more parallel pathways in the model may not capture oscillatory *M*(*t*). We then considered decomposing the basal pathway of the model (figure 1*d*) into multiple sequential steps, as shown in figure 4*a* for the cross-talking *n*-state model. The calculation of *M*(*t*) can follow the same procedure for the model of figure 1*d*, which relies on solving a system of differential equations for which the coefficient matrix may have multiple pairs of eigenvalues expressed by conjugate complexes (electronic supplementary material). According to the classical theory of ordinary differential equations, the exact forms of *M*(*t*) may contain multiple periodic cosine functions, and it is plausible to visualize oscillatory dynamics.

To test *M*(*t*) oscillation induced by the sequential multi-step gene activation, we compared the *M*(*t*) profiles for different step numbers. This procedure requires that all comparisons are restricted to a constant average duration *T*_off_ of the gene *off* state [10,37]. For the cross-talk *n*-state model (figure 4*a*), *T*_off_ is given by

$$T_{\mathrm{off}}=q_{1}\sum_{i=1}^{n-1}\frac{1}{\kappa_{i}}+\frac{q_{2}}{\lambda },\;n\geq 2.$$

To guarantee the unchanged *T*_off_, we set three parameter scaling conditions where activation rates *κ*_1_, …, *κ*_*n*−1_ are separately scaled identically [17,47], differently [8,17] and alternatively [17]

$$\begin{array}{rl} (C1)\;\;:\;\; & \kappa_{1}=\kappa_{2}=\cdots =\kappa_{n-1},\;\mathrm{with}\;\kappa_{1}=\frac{q_{1}(n-1)}{T_{\mathrm{off}}-q_{2}/\lambda };\\ (C2)\;\;:\;\; & \kappa_{2}=2\kappa_{1},\kappa_{3}=3\kappa_{1},\ldots \kappa_{n-1}=(n-1)\kappa_{1},\\ & \;\mathrm{with}\;\kappa_{1}=q_{1}\frac{1+(1/2)+\cdots +(1/\left(n-1\right))}{T_{\mathrm{off}}-q_{2}/\lambda };\\ (C3)\;\;:\;\; & \kappa_{1}=\kappa_{3}=\cdots ,\;\text{and}\;\kappa_{2}=\kappa_{4}=\cdots =3\kappa_{1},\\ & \;\mathrm{with}\;\kappa_{1}=\left\{ \begin{array}{c} \frac{2q_{1}(n-1)}{3(T_{\mathrm{off}}-q_{2}/\lambda )},\;n\;\text{is odd},\\ \frac{q_{1}(2n-1)}{3(T_{\mathrm{off}}-q_{2}/\lambda )},\;n\;\text{is even}. \end{array}\right. \end{array}$$

We initially examined the case of *q*_2_ = 0, for which a single multi-step pathway directs gene activation [10,17,27,28]. Under parameter scaling condition (C1), we generated several *M*(*t*) curves under different activation step numbers, using numerical simulations from the corresponding system of differential equations (electronic supplementary material, (19)). As shown in figure 4*b* under the fitted parameters of the *Cxcl1* gene in figure 2, multi-step gene activation for large step numbers triggered significantly damped oscillations *M*(*t*), where the significance of the oscillation is positively correlated with the step number. However, the system displayed lag times of more than 8 h to reach the first peak of *M*(*t*). This slow transcription response contradicts the rapid peak of *M*(*t*) within 0.5–2 h for mouse fibroblast genes [33,34]. Moreover, the damped oscillation and response lag of *M*(*t*) were robust against the parameter scaling conditions (C2) and (C3) (electronic supplementary material, figure S2). Collectively, the large number of sequential gene activation steps facilitates the oscillatory dynamics of *M*(*t*). At the same time, a single multi-step pathway could not induce quickly peaked transcription dynamics of mouse fibroblast genes.

We then quantified *q*_2_ ≠ 0 to introduce the cross-talking regulation of pathways on *M*(*t*) dynamics. We generated *M*(*t*) curves (electronic supplementary material, (18)) under the fitted parameters of the *Cxcl1* gene in figure 2 and conditions (C1)–(C3). As shown in figure 4*c* and electronic supplementary material, figure S3, the system generates oscillatory *M*(*t*) with two major features. Firstly, *M*(*t*) displays a quick and sharp first peak within the initial time region. In contrast, the height and sharpness of the first peak are nearly independent of the step number and parameter scaling conditions of the basal pathway. Secondly, *M*(*t*) displays a damped and gentle second and following peaks that are tightly correlated with the step number and parameter conditions, similarly to the oscillation of *M*(*t*) induced by a single multi-step pathway (figure 4*b*). These two features capture multiple dynamical peaks for the transcription of the mouse fibroblast *Nfkbia* gene [33] (figure 4*d*). Collectively, the cross-talk between signalling and basal pathways plays a dominant role in generating the first quick up-and-down transcription dynamics. By contrast, the subsequent gentle and damped transcription oscillation is induced by the multi-step regulation in the weak basal pathway.

### 2.5. Cross-verification with single-cell measurements

Our method requires rich *M*(*t*) data dynamics to rule out frameworks that cannot capture the dynamic features sufficiently. When *M*(*t*) data displays simple monotonic dynamics, all the gene activation frameworks in figure 1 may facilitate good fit to *M*(*t*) data. Therefore, we cannot rule out any activation framework by capturing the monotonic *M*(*t*) dynamics. To solve this dilemma, we may introduce some single-cell transcription data which display smooth trend lines along the timeline, such as the noise *CV*^2^(*t*), Fano factor *ϕ*(*t*) and probability *P*_0_(*t*) of the gene producing zero transcripts. These indexes may be used in conjunction with *M*(*t*) to cross-verify the gene activation frameworks [25].

The calculation of *ϕ*(*t*) and *CV*^2^(*t*) are standard [38,48], and require solving the second moment *μ*_2_(*t*) of the system of ordinary differential equations derived from the corresponding master equations [36,43]. We calculated the exact forms of *μ*_2_(*t*) for the cross-talking three-state model (electronic supplementary material, (24)), and *ϕ*(*t*) = *μ*_2_(*t*)/*M*(*t*) − *M*(*t*) and *CV*^2^(*t*) = *ϕ*(*t*)/*M*(*t*) were then readily obtained. The probability *P*_0_(*t*) is one of the solutions of master equations. The calculation of *P*_0_(*t*) involves introducing the generating function *V*(*z*, *t*) to transform the master equation into a system of partial differential equations for *V*(*z*, *t*). Although solving *V*(*z*, *t*) is somewhat difficult, we can still express *V*(*z*, *t*) in closed forms of hypergeometric functions for different models [23,28]. Consequently, *P*_0_(*t*) can be obtained by *P*_0_(*t*) = *V*( − 1, *t*). The expressions of *ϕ*(*t*), *CV*^2^(*t*) and *P*_0_(*t*) are not as neat as that of *M*(*t*), which prevents us from establishing theoretical bijections between their dynamical profiles and parameter regions. Nevertheless, the computation of *ϕ*(*t*), *CV*^2^(*t*) and *P*_0_(*t*) through their expressions or the corresponding differential equations (electronic supplementary material) can generate dynamical curves for fitting data [15,45].

**Figure 5.**
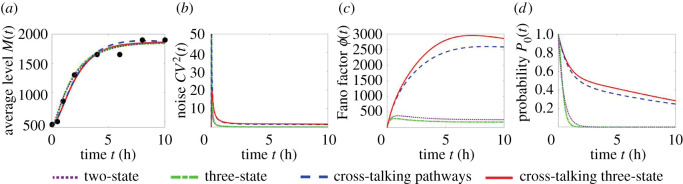
Discrimination of gene activation frameworks under monotonic dynamical mRNA average level. (*a*) The increasing transcription mean data (black circles, *Mmp13* gene [33]) are fitted by different models. (*b*) Dynamical curves of transcription noise for each model are clustered with relatively small values most of the time. For (*c*) transcription Fano factor and (*d*) probability of genes not producing any transcript, the dynamical curves generated by a single pathway deviate from the curves generated by cross-talking pathways. Parameters: *v* = 2630 h^−1^, *q*_2_ = 0.5; two-state model, (*λ*, *γ*, *δ*) = (2.21, 4, 0.5) h^−1^; three-state model, (*κ*_1_, *κ*_2_, *γ*, *δ*) = (5.09, 5.09, 4, 0.55) h^−1^; cross-talking pathways model, (*κ*, *λ*, *γ*, *δ*) = (0.07, 1.2, 1.2, 0.15) h^−1^; cross-talking three-state model, (*κ*_1_, *κ*_2_, *λ*, *γ*, *δ*) = (0.24, 0.1, 1.2, 1, 0.15) h^−1^.

We hypothesized that different gene activation frameworks can generate distinct dynamics of *ϕ*(*t*), *CV*^2^(*t*) and *P*_0_(*t*) under the same monotonic *M*(*t*). To verify this, we first fitted a group of increasing *M*(*t*) data to all four frameworks in figure 1 (figure 5*a*). Under the fitted parameters, we separately generated dynamical curves of *ϕ*(*t*), *CV*^2^(*t*) and *P*_0_(*t*) corresponding to each framework. The curves of noise *CV*^2^(*t*) clustered within a low-value region, probably because of their large denominator *M*(*t*) (figure 5*b*), suggesting that the noise data may not help discriminate gene activation frameworks. The curves of the Fano factor *ϕ*(*t*) are separated into two categories: the curves are very high under cross-talking pathways but are relatively low under a single pathway (figure 5*c*). This separation reinforces the conclusion that the parallel pathways enhance *ϕ* [43], while sequential steps suppress *ϕ* [10] under the same mean level *M* at steady state. The curves of *P*_0_(*t*) follow the same separation as that of *ϕ*(*t*) (figure 5*d*), which reinforces the observed suppressed *P*_0_(*t*) under multi-step gene activation [27]. Collectively, the dynamics of *ϕ*(*t*) and *P*_0_(*t*) may help discriminate the activation frameworks regulated by cross-talking pathways or a single pathway.

## 3. Conclusion and discussion

The classical two-state model used in single-cell studies posits that a gene will randomly transition between *on* (active) and *off* (inactive) states, with mRNA molecules being only produced when the gene is *on* (figure 1*a*). Compared to the universal feature of a single rate-limiting step turning gene *off* [6,8,9], the process of turning a gene *on* is typically influenced by multiple rate-limiting fluctuations [6,7,9]. The framework of gene activation has been modelled by listing rate-limiting steps sequentially [4,8,17,29] (figure 1*b*), in parallel [11,38,39] (figure 1*c*), or in the form of their combinations [12] (figure 1*d*). These activation frameworks, coupled with simple gene inactivation, mRNA birth and death processes, lead to different mathematical models of stochastic gene transcription (figure 1).

Recent studies have facilitated efficient computational method for fitting the transcription distribution data to search the optimal number of rate-limiting steps in gene activation and their kinetic parameters [13,17,20]. The workflow of these methodologies typically starts with generalized chemical master equations for which a single kinetic rate controls each reaction [13,25]. This assumption implicitly suggests a single pathway with sequential rate-limiting steps directing the gene activation (figure 1*a*,*b*), while ignores cross-talking parallel pathways (figure 1*c*,*d*) for which the kinetic rates in master equations may be multiplied by pathway selection probabilities *q*_1_ and *q*_2_ (equations (2.1) and (2.2)).

We noticed several controversial conclusions on the gene activation obtained by fitting transcription distribution data. For example, using the two-state model (figure 1*a*) led to an acceptable fit to dynamical mRNA distribution data for mammalian *c-Fos* gene [21]. However, the mRNA average level *M*(*t*), extracted from the distribution data, exhibited a significant up-and-down dynamics [21] which cannot be captured by the two-state model as it only generates increasing *M*(*t*) (table 1). By contrast, the cross-talking pathway model (figure 1*c*) can generate up-and-down *M*(*t*) (table 1) and thus may serve as a better candidate model for the *c-Fos* gene. Another controversy arose from unimodal distribution data of *off* duration for 16 mouse fibroblast genes which have been fitted by the three-state model (figure 1*b*) [8]. However, the three-state model may not be appropriate as it fails to capture non-monotonic dynamical *M*(*t*) data (table 1) for a large number of mouse fibroblast genes [33,34], whereas the cross-talking three-state model (figure 1*d*) provides good fit to those *M*(*t*) data (figure 2*d*). Therefore, it is crucial to determine the confidential gene activation framework in parallel pathways and multiple steps before attempting to fit single-cell dynamical transcription data computationally.

This study demonstrated that the transcript average level *M*(*t*) is competent for prior estimation of gene activation framework. Compared to the multiple uneven mRNA distribution profiles at discrete time points [13,20], a single smooth curve of *M*(*t*) along the timeline presents easily discriminated dynamic features. Subsequently, frameworks that cannot capture the exhibited *M*(*t*) dynamical features can be ruled out, while the other potential frameworks are further determined by fitting *M*(*t*) data. The calculation of *M*(*t*) and its fitting to the data under arbitrary initial conditions (equation (2.7)) are standard using ordinary differential equations [44] or master equations [25]. The challenge is determining whether the considered framework can or cannot display the exhibited *M*(*t*) features. Solving this challenge requires rigorous but tedious mathematical analysis to establish bijection theory (table 1, appendix theorems A.1 and A.2) that shows one-to-one correspondence between parameter regions and *M*(*t*) dynamical features for each activation framework of the model. We noticed that the initial condition of the system could significantly influence *M*(*t*) dynamics [49]. Here we restricted our analysis under the condition of zero transcripts at time *t* = 0 (equation (2.11)), consistent with the basal expression level of genes under normal cellular growth conditions before adding external inductions [15,21,33].

We illustrated our idea using *M*(*t*) for many TNF-induced mouse fibroblast genes. These genes display rich transcription dynamics that can be categorized into three main features: increasing, up-and-down and up-down-up profiles of *M*(*t*) [33,34]. Our bijection theories (table 1 and appendix theorems A.1 and A.2) show that these distinct dynamics cannot be achieved by the sequential or parallel rate-limiting steps alone but can be captured by the simplest form of combined sequential and parallel steps (figure 2). We call this the cross-talking three-state framework, as depicted in figure 1*d*, for which the gene is activated either by the weak basal pathway consisting of two sequential steps or by the strong signalling pathway. Furthermore, analysis of the freely fitted parameters of the cross-talking three-state model reveals regulation scenarios of combined gene activation, inactivation and mRNA degradation to navigate monotonicity of *M*(*t*) dynamics (figure 3; electronic supplementary material, figure S1 and table S1). These observations may facilitate answering the proposed question in [33] of how the transcriptional control cooperates with transcript stability to determine the kinetics of TNF-induced gene expression.

We note that a small number of mouse fibroblast genes display damped transcription oscillation, with the first peak forming rapidly within the initial period [33,34]. We first ruled out the widely used framework of a single multi-step pathway as it triggers a long lag reaching the first peak of *M*(*t*) (figure 4*b*; electronic supplementary material, figure S2), which contradicts the observed rapid peak of *M*(*t*). Also, the cross-talking three-state framework cannot generate oscillatory dynamics of *M*(*t*) (appendix theorems A.1 and A.2). However, when we developed a cross-talking *n*-state framework by decomposing the basal pathway of a cross-talking three-state framework into multiple steps (figure 4*a*), the oscillation appeared, and the first peak of *M*(*t*) formed quickly (figure 4*c*; electronic supplementary material, figure S3). Together with the good fit to transcription data (figure 4*d*), we confirmed that the cross-talk between pathways is crucial to trigger the first rapid, sharp peak of *M*(*t*), while the multi-step regulation facilitates the following damped and gentle oscillatory dynamics, for mouse fibroblast genes.

Our conclusions suggested that all the representative datasets on *M*(*t*) dynamics of mouse fibroblast genes can be universally fitted by the cross-talking *n*-state (*n* ≥ 3) activation framework. By contrast, the other simpler frameworks fail to capture several *M*(*t*) dynamical features. The cross-talking *n*-state framework may also describe similar non-monotonic transcription dynamics of *c-Fos* genes after serum induction [21] or the genes in the innate immune system of insects when fighting pathogen invasions [50]. Therefore, before the fit of sophisticated distribution data for those genes on *off* duration or mRNA copy numbers, the cross-talking *n*-state framework should be introduced into the master equations as in equations (2.1) and (2.2). Otherwise, the computational methods may only estimate optimal parameters and step numbers using an inappropriate gene activation framework.

Our procedure relies on the rich dynamics of *M*(*t*). When *M*(*t*) behaves monotonically, the other transcription indexes at single-cell level are required to cross-verify the activation frameworks. For instance, the dynamical Fano factor or the probability of no transcript being produced may help discriminate frameworks regulated by a single pathway or cross-talking pathways (figure 5). Future work may use additional data for different genes and transcription indexes to test and develop our procedure. In addition, we noted the masking effect of small mRNA degradation rate on *M*(*t*)-rich dynamics (figure 3*b*). We anticipate the inclusion of nascent RNA data that are not influenced by the mRNA degradation rate and may therefore provide more direct information on gene activation frameworks [4,51]. Finally, a cell cycle description [22,47] may be introduced into gene activation frameworks to eliminate estimation errors caused by disregarding cell cycle stochasticity [47].

## Data accessibility

All scripts used in this study are openly accessible through https://github.com/StochasticBiology/boolean-efflux.git. The data are provided in electronic supplementary material [52].

## Appendix A. Mathematical analysis of *M*(*t*) dynamics for cross-talking three-state model

In this section, we use exact forms (2.8)–(2.10) of *M*(*t*) to understand its dynamical features. We consider the initial condition (2.11) for which the gene is silent, and there is almost no transcript at the initial time [15,21,33]. The following two theorems illustrate the cases of $c_{1}^{2}>c_{2}$ and $c_{1}^{2}<c_{2}$, respectively.

Theorem A.1. *For arbitrary non-zero real numbers* *a*, *b*, *c* *and positive real numbers* *M**, *α*, *β*, *r*, *if* *M*(*t*) *is expressed as*

$$M(t)=M^{*}+ae^{-\alpha t}+be^{-\beta t}+ce^{-rt}\geq 0,\;0<\alpha <\beta <r,\;M(0)=0,\;\text{and}\;M^{\prime }(0)\geq 0,$$

*then* *M*(*t*) *displays only one of increasing, up-and-down and up-down-up dynamical profiles*.

Proof. The derivative of *M*(*t*) gives

A 1 $$M^{\prime }(t)=-\alpha ae^{-\alpha t}-\beta be^{-\beta t}-rce^{-rt}\;⟹\;M^{\prime }(t)e^{rt}=-[rc+\alpha ae^{(r-\alpha )t}+\beta be^{(r-\beta )t}].$$

We divided the discussion into two cases of *a* > 0 and *a* < 0.

(I) If *a* > 0, lim _*t*→∞_*M*′(*t*) < 0 by noting that the sign of *M*′(*t*) is dominated by the term ‘−*αa*e^−*αt*^’ when *t* → ∞. Taking the derivative of *M*′(*t*)e^*rt*^ in (A 1) gives
  A 2 $${\left[M^{\prime }(t)e^{rt}\right]}^{\prime }=-\alpha a(r-\alpha )e^{(r-\alpha )t}M_{1}(t),\;\mathrm{with}\;M_{1}(t)=1+\frac{\beta b(r-\beta )}{\alpha a(r-\alpha )}e^{(\alpha -\beta )t}.$$
  If *b* ≥ −*αa*(*r* − *α*)/*β*(*r* − *β*), (A 2) indicates
  $$M_{1}(t)>0\;⟹\;{\left[M^{\prime }(t)e^{rt}\right]}^{\prime }<0\;⟹\;M^{\prime }(t)e^{rt}\;\mathrm{decreases},\;\mathrm{for}\;t>0.$$
  There are two probabilities of *M*′(0) > 0 and *M*′(0) = 0. If *M*′(0) > 0, together with lim _*t*→∞_*M*′(*t*) < 0, there exists *t*_1_ > 0 such that
  $$M^{\prime }(t)e^{rt}>0,\;\mathrm{for}\;t\in (0,t_{1}),\;M^{\prime }(t_{1})e^{rt_{1}}=0\;\text{and}\;M^{\prime }(t)e^{rt}<0,\;\mathrm{for}\;t>t_{1}.$$
  Therefore, *M*(*t*) increases for *t* ∈ (0, *t*_1_) and decreases for *t* > *t*_1_ which presents up-and-down dynamics. If *M*′(0) = 0, *M*′(*t*)e^*rt*^ < 0 for *t* > 0, and thus *M*(*t*) decreases with *M*(*t*) < 0 all the time. This contradicts to the assumption of *M*(*t*) ≥ 0.
  If *b* < −*αa*(*r* − *α*)/*β*(*r* − *β*), (A 2) suggests that there is *t*_2_ > 0 such that
  $$\begin{array}{rl} & M_{1}(t)<0,\;t\in (0,t_{2}),\;M_{1}(t_{2})=0\;\text{and}\;M_{1}(t)>0,t>t_{2},\;\text{where}\\ & t_{2}=\frac{1}{\alpha -\beta }\ln\left(\frac{-a\alpha (r-\alpha )}{b\beta (r-\beta )}\right). \end{array}$$
  Then, (A 2) suggests that [*M*′(*t*)e^*rt*^]′ > 0 for *t* ∈ (0, *t*_2_) and [*M*′(*t*)e^*rt*^]′ < 0 for *t* > *t*_2_. Therefore, *M*′(*t*)e^*rt*^ increases in (0, *t*_2_) while decreases in (*t*_2_, ∞). As *M*′(0) ≥ 0 and lim _*t*→∞_*M*′(*t*) < 0, there exists *t*_3_ such that $M^{\prime }(t_{3})e^{rt_{3}}=0$. Thus *M*′(*t*)e^*rt*^ > 0 for *t* ∈ (0, *t*_3_) and *M*′(*t*)e^*rt*^ < 0 for *t* > *t*_3_. This indicates that *M*(*t*) increases for *t* ∈ (0, *t*_3_) and decreases for *t* > *t*_3_, which is the up-and-down dynamics.
(II) If *a* < 0, the (A 1) indicates that lim _*t*→∞_*M*′(*t*) > 0. If *b* ≤ −*αa*(*r* − *α*)/*β*(*r* − *β*), (A 2) gives
  $$M_{1}(t)>0\;⟹\;{\left[M^{\prime }(t)e^{rt}\right]}^{\prime }>0\;⟹\;M^{\prime }(t)e^{rt}\;\mathrm{increases},\;\mathrm{for}\;t>0.$$
  As *M*′(0) ≥ 0, we have *M*′(*t*)e^*rt*^ ≥ 0, *t* > 0 which suggests that *M*(*t*) increases for all the time.

Contrastingly, if *b* > −*αa*(*r* − *α*)/*β*(*r* − *β*), from (A 2) we find that there exists *t*_4_ > 0 such that

$$\begin{array}{rl} & M_{1}(t)<0,\;t\in (0,t_{4}),\;M_{1}(t_{4})=0\;\text{and}\;M_{1}(t)>0,\;t>t_{4},\;\mathrm{where}\\ & t_{4}=\frac{1}{\alpha -\beta }\ln\left(\frac{-a\alpha (r-\alpha )}{b\beta (r-\beta )}\right). \end{array}$$

Together with (A 2), this further leads to [*M*′(*t*)e^*rt*^]′ < 0 for *t* ∈ (0, *t*_4_) and [*M*′(*t*)e^*rt*^]′ > 0 for *t* > *t*_4_. Therefore, *M*′(*t*)e^*rt*^ decreases in (0, *t*_4_) while increases in (*t*_4_, ∞). As *M*′(0) ≥ 0, there are two possibilities of $M^{\prime }(t_{4})e^{rt_{4}}\geq 0$ and $M^{\prime }(t_{4})e^{rt_{4}}<0$. For $M^{\prime }(t_{4})e^{rt_{4}}\geq 0$, the fact of lim _*t*→∞_*M*′(*t*) > 0 indicates *M*′(*t*)e^*rt*^ ≥ 0 for all *t* ≥ 0 and thereby *M*(*t*) increases all the time. If it is $M^{\prime }(t_{4})e^{rt_{4}}<0$, there exists 0 < *t*_5_ < *t*_4_ < *t*_6_ such that $M^{\prime }(t_{5})e^{rt_{5}}=M^{\prime }(t_{6})e^{rt_{6}}=0$ and

$$M^{\prime }(t)e^{rt}>0,\;t\in (0,t_{5}),\;M^{\prime }(t)e^{rt}<0,\;t\in (t_{5},t_{6})\;\text{and}\;M^{\prime }(t)e^{rt}>0,\;t>t_{6}.$$

This indicates that *M*(*t*) displays an up-down-up dynamics that *M*(*t*) increases for $t\in (0,t_{5})\cup (t_{6},\infty )$ and decreases within *t* ∈ (*t*_5_, *t*_6_). The proof is completed. ▪

Theorem A.2. *Let condition* $c_{1}^{2}<c_{2}$ *hold. Let*

A 3 $$H(x)=vh(x)=vq_{2}\lambda x^{2}+v[q1\kappa_{1}\kappa_{2}+q2\lambda (\kappa_{1}+\kappa_{2})]x+v\kappa_{1}\kappa_{2}\lambda ,$$

*and* *x*_1_ *and* *x*_2_ *be two roots of* *H*(*x*). *Without loss of generality, assume* *δ* ≠ *a*_1_, *a*_2_, *a*_3_ *given by electronic supplementary material, (11)*. *If* *x*_1_ *and* *x*_2_ *are complex valued, either* *M*(*t*) *increases monotonically for all* *t* > 0, *or* *M*(*t*) *develops up-down-up dynamics. If* *x*_1_ *and* *x*_2_ *are real valued and* *x*_1_ < *x*_2_, *we have*:

(1) *If one of the following occurs*: (i) −*x*_2_ < {*a*_1_, *a*_2_, *a*_3_, *δ*} < −*x*_1_, (ii) −*x*_2_ < {*a*_1_, *a*_2_, *δ*} < −*x*_1_ < *a*_3_, (iii) −*x*_2_ < {*a*_1_, *δ*} < −*x*_1_ < *a*_2_, (iv) −*x*_2_ < *a*_1_ < *a*_2_ < *a*_3_ < −*x*_1_ < *δ*, (v) −*x*_2_ < *a*_1_ < *a*_2_ < −*x*_1_ < min{*a*_3_, *δ*}, (vi) −*x*_2_ < *a*_1_ < −*x*_1_ < min{*a*_2_, *δ*}, (vii) −*x*_2_ < *δ* < −*x*_1_ < *a*_1_, *m*(*t*) *increases initially until reaching a peak and then goes down (up-and-down)*.
(2) *If one of the following occurs*: (i) *a*_1_ < −*x*_2_ < *a*_2_ < *a*_3_ < −*x*_1_ < *δ*, (ii) *a*_1_ < −*x*_2_ < *a*_2_ < *a*_3_ < −*x*_1_ < *δ*, (iii) *a*_1_ < −*x*_2_ < *a*_2_ < −*x*_1_ < min{*a*_3_, *δ*}, (iv) *a*_1_ < −*x*_2_ < {*a*_2_, *δ*} < −*x*_1_ < *a*_3_, (v) max{*a*_1_, *δ*} < −*x*_2_ < *a*_2_ < −*x*_1_ < *a*_3_, (vi) *δ* < −*x*_2_ < *a*_1_ < *a*_2_ < −*x*_1_ < *a*_3_, (vii) *a*_1_ < −*x*_2_ < *δ* < −*x*_1_ < *a*_2_, *and* (viii) *δ* < −*x*_2_ < *a*_1_ < −*x*_1_ < *a*_2_, *M*(*t*) *increases monotonically for all* *t* > 0.
(3) *If one of the following occurs*: (i) −*x*_1_ < min{*a*_1_, *δ*}, (ii) *a*_1_ < −*x*_2_ < −*x*_1_ < min{*a*_2_, *δ*}, (iii) *a*_2_ < −*x*_2_ < −*x*_1_ < min{*a*_3_, *δ*}, (iv) *a*_3_ < −*x*_2_ < −*x*_1_ < *δ*, (v) max{*a*_1_, *δ*} < −*x*_2_, (vi) max{*a*_2_, *δ*} < −*x*_2_ < −*x*_1_ < *a*_3_, (vii) max{*a*_3_, *δ*} < −*x*_2_, (viii) *δ* < −*x*_2_ < −*x*_1_ < *a*_1_, (ix) *a*_3_ < −*x*_2_ < *δ* < −*x*_1_, (x) *a*_2_ < −*x*_2_ < *a*_3_ < −*x*_1_ < *δ*, (xi) *a*_2_ < −*x*_2_ < {*a*_3_, *δ*} < −*x*_1_, (xii) {*a*_2_, *δ*} < −*x*_2_ < *a*_3_ < −*x*_1_, (xiii) *a*_1_ < −*x*_2_ < {*a*_2_, *a*_3_, *δ*} < −*x*_1_, (xiv) {*a*_1_, *δ*} < −*x*_2_ < *a*_2_ < *a*_3_ < −*x*_1_, (xv) *δ* < −*x*_2_ < *a*_1_ < *a*_2_ < *a*_3_ < −*x*_1_, *either* *M*(*t*) *increases monotonically for all* *t* > 0, *or* *M*(*t*) *develops up-down-up dynamical profile*.

Proof. The substitution of the initial condition (2.11) into the differential system (electronic supplementary material, (11)) gives *M*′(0) = 0, *P*_*e*_′(0) > 0, and in turn, *M*″(0) = *v P*_*e*_′(0) > 0. Consequently, *M*(*t*), *M*′(*t*) and *M*″(*t*) are all positive for *t* > 0 sufficiently small. The initial values (2.11) simplify the exact form of *M*(*t*) (equation (2.9) of main text) in the form of

$$M(t)=\frac{v}{\delta }\cdot \frac{\kappa_{1}\kappa_{2}\lambda }{a_{1}a_{2}a_{3}}-\frac{\beta_{1}}{a_{1}}e^{-a_{1}t}-\frac{\beta_{2}}{a_{2}}e^{-a_{2}t}-\frac{\beta_{3}}{a_{3}}e^{-a_{3}t}-\frac{\beta_{\delta }}{\delta }e^{-\delta t},$$

where

$$\beta_{1}=\frac{H(-a_{1})}{\left(\delta -a_{1})(a_{2}-a_{1})(a_{3}-a_{1}\right)},\;\beta_{2}=\frac{H(-a_{2})}{\left(\delta -a_{2})(a_{1}-a_{2})(a_{3}-a_{2}\right)}$$

and

$$\beta_{3}=\frac{H(-a_{3})}{\left(\delta -a_{3})(a_{1}-a_{3})(a_{2}-a_{3}\right)},\;\beta_{\delta }=\frac{H(-\delta )}{\left(a_{1}-\delta )(a_{2}-\delta )(a_{3}-\delta \right)}.$$

Thus

A 4 $$M^{\prime }(t)=\beta_{1}e^{-a_{1}t}+\beta_{2}e^{-a_{2}t}+\beta_{3}e^{-a_{3}t}+\beta_{\delta }e^{-\delta t}.$$

We first consider the situation of complex valued *x*_1_ and *x*_2_. There are total four parameter cases and we only present the proof for the case *δ* < *a*_1_ < *a*_2_ < *a*_3_, as the other cases *a*_1_ < *δ* < *a*_2_ < *a*_3_, *a*_1_ < *a*_2_ < *δ* < *a*_3_ and *a*_1_ < *a*_2_ < *a*_3_ < *δ* can be treated similarly. If *x*_1_ and *x*_2_ are complex valued, *H*(*x*) > 0 for all *x*. In the view of *δ* < *a*_1_ < *a*_2_ < *a*_3_, we obtain

A 5 $$\beta_{1}<0,\;\beta_{2}>0,\;\beta_{3}<0\;\text{and}\;\beta_{\delta }>0.$$

For *t* > 0 sufficiently large, *M*′(*t*) is dominated by *β*_*δ*_exp ( − *δt*) in (A 4) and is therefore positive. It follows from (A 4) that

A 6 $$\begin{array}{rl} & {\left[e^{a_{3}t}M^{\prime }(t)\right]}^{\prime }=e^{(a_{3}-\delta )t}g(t),\\ & \;\mathrm{with}\;g(t)=(a_{3}-\delta )\beta_{\delta }+(a_{3}-a_{1})\beta_{1}e^{(\delta -a_{1})t}+(a_{3}-a_{2})\beta_{2}e^{(\delta -a_{2})t}. \end{array}$$

It is seen that

A 7 $$g(0)=M^{\prime \prime }(0)>0\;\mathrm{and}\;\;\lim_{t\to \infty }g(t)=(a_{3}-\delta )\beta_{\delta }>0.$$

We can see from (A 6) that

A 8 $$M^{\prime }(t)=e^{-a_{3}t}G(t),\;\mathrm{where}\;G(t)=\int_{0}^{t}e^{(a_{3}-\delta )s}g(s)ds,\;t>0.$$

It follows from (A 4) and (A 8) that

A 9 $$G(0)=0,\;\lim_{t\to \infty }G(t)=+\infty \;\text{and}\;G^{\prime }(t)=e^{(a_{3}-\delta )t}g(t).$$

Differentiating *g*(*t*) gives

$$g^{\prime }(t)=\frac{H(-a_{1})}{a_{2}-a_{1}}e^{(\delta -a_{2})t}\left[e^{(a_{2}-a_{1})t}-\frac{H(-a_{2})}{H(-a_{1})}\right].$$

If *H*( − *a*_2_) ≤ *H*( − *a*_1_), *g*′(*t*) ≥ 0, and so *g*(*t*) > 0 for all *t* > 0. Hence (A 8) gives *M*′(*t*) > 0 for all *t* > 0. If *H*( − *a*_2_) > *H*( − *a*_1_), *g*′(*t*) < 0 in (0, *t*_0_) for

$$t_{0}=\frac{1}{a_{2}-a_{1}}[\ln(H(-a_{2}))-\ln(H(-a_{1}))],$$

and becomes positive for *t* > *t*_0_. If *g*(*t*_0_) ≥ 0, *g*(*t*) > 0 for all *t* > 0 and *t* ≠ *t*_0_. Thus (A 8) gives again *M*′(*t*) > 0 for all *t* > 0. If *g*(*t*_0_) < 0, (A 7) implies that *g*(*t*) has two zeros *t*_1_ > 0 and *t*_2_ > 0 with *g*(*t*) > 0 in $\left(0,t_{1})\cup (t_{2},+\infty \right)$, and *g*(*t*) < 0 in (*t*_1_, *t*_2_). It follows from (A 9) that

A 10 $$G^{\prime }(t)>0\;\mathrm{for}\;t\in (0,t_{1})\cup (t_{2},+\infty )\;\text{and}\;G^{\prime }(t)<0\;\mathrm{for}\;t\in (t_{1},t_{2}).$$

If *G*(*t*_2_) ≥ 0, *G*(*t*) > 0 for all *t* > 0 and *t* ≠ *t*_2_, and so *M*′(*t*) > 0 for all *t* > 0 and *t* ≠ *t*_2_. We recall that *G*(*t*) > 0 for *t* > 0 both sufficiently small and large. Thus, if *G*(*t*_2_) < 0, (A 10) implies that *G*(*t*) has two zeros *t*_3_ > 0 and *t*_4_ > 0 with *G*(*t*) > 0 in $\left(0,t_{3})\cup (t_{4},+\infty \right)$, and *G*(*t*) < 0 in (*t*_3_, *t*_4_). It follows from (A 8) that *M*′(*t*) > 0 in $\left(0,t_{3})\cup (t_{4},+\infty \right)$ and *M*′(*t*) < 0 in (*t*_3_, *t*_4_). This finishes the proof of the case when *x*_1_ and *x*_2_ are complex valued. In the following, we assume that *x*_1_ and *x*_2_ are real valued.

(1) We present the proof with the additional assumption that −*x*_2_ < *a*_1_ < *a*_2_ < *δ* < −*x*_1_ < *a*_3_, as the other cases can be dealt with by the same idea. As
  A 11 $$-x_{2}<a_{1}<a_{2}<\delta <-x_{1}<a_{3}\Rightarrow \beta_{1}<0,\beta_{2}>0,\beta_{3}<0,\;\text{and}\;\beta_{\delta }<0,$$
  using (A 4) again, we obtain
  A 12 $$\begin{array}{rl} & {\left[e^{a_{2}t}M^{\prime }(t)\right]}^{\prime }=e^{(a_{2}-a_{1})t}g_{1}(t),\\ & \;g_{1}(t)=(a_{2}-a_{1})\beta_{1}+(a_{2}-\delta )\beta_{\delta }e^{(a_{1}-\delta )t}+(a_{2}-a_{3})\beta_{3}e^{(a_{1}-a_{3})t}, \end{array}$$
  with *f*(0) = *M*″(0) > 0 and lim _*t*→∞_*g*_1_(*t*) = (*a*_2_ − *a*_1_)*β*_1_ < 0. In terms of (A 11) and (A 12), we have (*a*_2_ − *δ*)(*a*_1_ − *δ*)*β*_*δ*_ < 0 and (*a*_2_ − *a*_3_)(*a*_1_ − *a*_3_)*β*_3_ < 0, and so
  $$f^{\prime }(t)=(\delta -a_{2})(a_{1}-a_{2})\beta_{2}e^{(\delta -a_{2})t}+(\delta -a_{3})(a_{1}-a_{3})\beta_{3}e^{(\delta -a_{3})t}<0.$$
  Hence, there exists a *t*_5_ > 0 such that *g*_1_(*t*) > 0 in (0, *t*_5_), *g*_1_(*t*_5_) = 0, and *g*_1_(*t*) < 0 in (*t*_5_, + ∞). For *t* > 0 sufficiently large, *M*′(*t*), dominated by the term with exp ( − *a*_1_) in (A 4), is negative. We also recall that *M*′(*t*) > 0 for *t* > 0 sufficiently small. Thus, there exists a finite *t*_6_ > 0 such that *M*′(*t*) > 0 in (0, *t*_6_), and *M*′(*t*_6_) = 0. Then, *M*″(*t*_6_) ≤ 0, and (A 12) gives *f*(*t*_6_) = exp (*a*_1_ *t*_6_)*M*″(*t*_6_) ≤ 0. It follows that *t*_6_ ≥ *t*_5_ and *f*(*t*) < 0 for all *t* > *t*_6_. Hence, by using (A 12) again, for each *t* > *t*_6_, we have
  $$M^{\prime }(t)=e^{-a_{2}t}\int_{t_{6}}^{t}e^{(a_{2}-\delta )s}f\;(s)ds<0.$$
(2) We present the proof for the case *a*_1_ < *a*_2_ < −*x*_2_ < *δ* < −*x*_1_ < *a*_3_, as the other cases can be handled similarly. With this specification, we have
  A 13 $$\beta_{1}>0,\;\beta_{2}<0,\;\beta_{3}<0\;\text{and}\;\beta_{\delta }<0.$$
  By (A 4) and (A 13), we obtain for all *t* > 0
  $${\left[e^{a_{1}t}M^{\prime }(t)\right]}^{\prime }=(a_{1}-\delta )\beta_{\delta }e^{(a_{1}-\delta )t}+(a_{1}-a_{2})\beta_{2}e^{(a_{1}-a_{2})t}+(a_{1}-a_{3})\beta_{3}e^{(a_{1}-a_{3})t}>0.$$
  As *M*′(0) = 0, it shows that $e^{a_{2}t}M^{\prime }(t)>0$, and therefore, *M*′(*t*) > 0 for all *t* > 0.
(3) We give a short description for the proof with the additional condition that *x*_1_ < *x*_2_ < *δ* < *a*_1_ < *a*_2_ < *a*_3_, as the other cases can be proceeded in an analogous manner. Under this extra condition, (A 5) holds again. Thus, the remaining discussion is the same as the proof given above for two complexes valued *x*_1_ and *x*_2_, and we omit it here.

 ▪
